# Gender representation at scientific congresses: focus on functional and female urology—a study from the EAU Young Academic Urologist Functional Urology Group

**DOI:** 10.1007/s00345-023-04355-6

**Published:** 2023-03-21

**Authors:** Tanja Hüsch, Nadir I. Osman, François Herve, Mehmet G. Culha, Luís Vale, Antonio Tienza, Manuela Tutolo, Sabrina De Cillis, Cyrille Guillot-Tantay, Véronique Phé, the EAU Young Academic Urologist Functional Group

**Affiliations:** 1 Department of Urology and Pediatric Urology, University Medical Center of Johannes Gutenberg University, Langenbeckstr. 1, 55131 Mainz, Germany; 2 Royal Hallamshire Hospital, Sheffield, UK; 3 Department of Urology, Ghent University Hospital, Ghent, Belgium; 4 University of Health Sciences, Prof. Dr. Cemil Tascioglu City Hospital, Istanbul, Turkey; 5 Department of Urology, Centro Hospitalar Universitário São João, Porto, Portugal; 6 Urology Department, Hospital Universitari Son Espases, Palma, Spain; 7 Division of Oncology, Unit of Urology, Urological Research Institute, IRCCS Ospedale San Raffaele, Milan, Italy; 8 Division of Urology, Department of Oncology, San Luigi Gonzaga Hospital, University of Turin, Orbassano, TO Italy; 9 Hôpital Foch, Service d’Urologie, Suresnes, France; 10 Department of Urology, Sorbonne University, Assistance Publique-Hôpitaux de Paris, Tenon Academic Hospital, Paris, France

**Keywords:** Female urology, Functional urology, Gender gap, Urology, Women

## Abstract

**Purpose:**

Female representation at scientific conferences is crucial for encouraging women pursuing an academic career. Nevertheless, gender inequity at urological conferences is common place and women are often stereotyped choosing functional urology. However, there is no evidence whether female representation is higher in functional urology. This investigations aims to analyze gender representation at functional urology sessions.

**Methods:**

National and international urological congresses between 2019 and 2021 with a focus on functional urology and female urology sessions were evaluated. Congresses were categorized as national or international. Session type, topic, gender of chairs and speakers of the identified sessions were recorded. In addition, affiliation and medical specialty were collected for chairs.

**Results:**

A total of 29 congresses were evaluated. Out of a total of 2893 chairs and speakers, 1034 (35.7%) were women and 1839 (63.6%) were men. This represents an overall gender gap of 27.9% for functional urology sessions. No significant differences in gender representation between national and international congresses could be identified (*p = *0.076). When considering gender distribution of chairs, the gap was more pronounced by 35.5%. Furthermore, men were more likely to be invited to be a speaker in plenary and podiums sessions.

**Conclusions:**

Gender inequality is present in functional urology sessions. There is a need for greater efforts to achieve gender equality. An important step to remedy the situation is the inclusion of women in scientific program committees. Furthermore, support by the leadership of urological societies and academic departments is essential to herald a lasting change in gender inequality.

## Introduction

Involvement in academic conferences is an important gateway to an academic career and continues to be routine part of an academic career path [1]. The presence of female role models correlates with increase positive self-conception and reduced stereotype application to young female scientists [2]. Nevertheless, women are often underrepresented at academic conferences [3] despite the increasing number of female physicians [4, 5]. The perception of female underrepresentation in academic environments may have substantial impact on the outcomes for women in academic medicine and may even contribute to women exiting an academic career [1]. A contributory factor may be that men do not perceive sexism in the same way as women. Consequently, they may doubt the negative experience of women which leads to further marginalization [1]. Such the denial of the existence of sexism leads perpetuates discrimination and serves to maintain the status quo in practices and policies [6]. This is a viscous cycle which further undermines women and continues to put them at a disadvantage in this environment.

The degree of female representation and how welcoming such meetings are to women is also dependent on the disciplines addressed and content of conferences [1]. In this context, surgical disciplines have been identified to be particularly affected by underrepresentation of women although differences exist by surgical specialty [7]. In urology, a gender gap of 65% for a large European conference has been identified [4] which has been confirmed by another analysis of gender representation of other urological meetings [8]. However, the International Continence Society has been identified to represent a more balanced gender representation [8]. This might be contributed by the higher proportion of women specializing in incontinence and female urology and by multidisciplinary nature of this meeting, including representation from nursing and allied healthcare professionals [9]. However, there is no specific evidence addressing whether female representation is higher in functional urology and female urology.

The current study is at assessing contemporary gender representation in functional urology and female urology sessions through an analysis of various national and international congresses and scientific meetings.

## Materials and methods

National and international urological congresses between 2019 and 2021 with focus on the topics of functional urology and female urology were evaluated. No ethical vote was required because no human being or animal was involved in this study. Selected were national and international urological and/or urogynaecological and/or neurourological congresses. Only sessions targeting functional urology and female urology were included. If international congresses were evaluated, the host country was recorded. Congresses in Belgium, France, Germany, Italy, Portugal, Turkey, Spain, United Kingdom, Sweden, Switzerland, Australia and United States were evaluated. Congresses were categorized in national and international types. Session type, topic and gender of chairs and speakers of the identified sessions were recorded. In addition, affiliation and medical specialty (urology vs. other) were collected for chairs. Descriptive analysis was applied. Univariate analysis to identify correlations between gender and the collected variables was performed. The Cochrane–Mantel–Haenszel test was used to assess independence of categorical predictors associated with congress type. A *p* value < 0.05 was considered significant. Statistical analysis was performed using SPSS 27 (IBM, Armonk, United States).

## Results

A total of 29 national and international congresses in 12 different European countries, and two congresses in the United States and Australia, were evaluated. A total of 2893 chairs and speakers were available for analysis. Of these, 1034 (35.7%) were women and 1839 (63.6%) were men, whereas 20 (0.7%) gender were not identifiable. This represents an overall gender gap of 27.9%. No significant differences in gender representation between national and international congresses could be identified (*p = *0.076).

### Chair analysis

A total of 758 chairs were identified in functional and female urology topics, distributed by 357 (47.1%), 131 (17.3%) and 270 (35.6%) for the years of 2019, 2020 and 2021, respectively. 482 (63.3%) chairs were represented in national and 276 (36.4%) in international congresses. Gender distribution was 513 (67.7%) and 244 (32.2%) for male and female chairs, respectively. One (0.1%) gender reference was missing. No significant difference of gender distribution between the years could be identified (*p = *0.465). This indicates a pooled gender gap for chairs in functional urology of 35.5%.

**Fig. 1:**
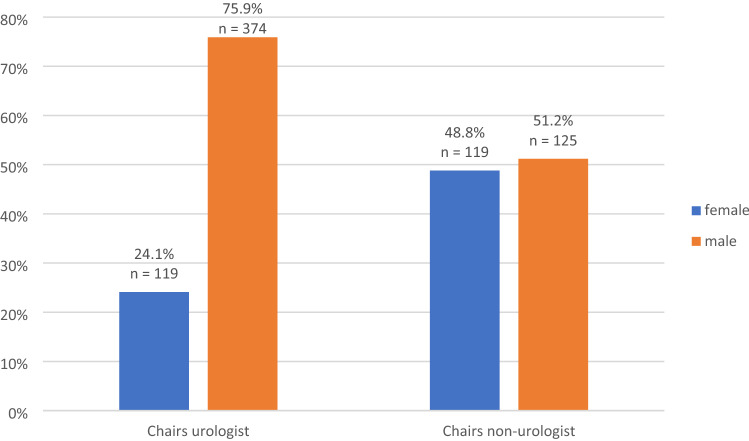
Chair gender distribution by medical speciality

**Fig. 2:**
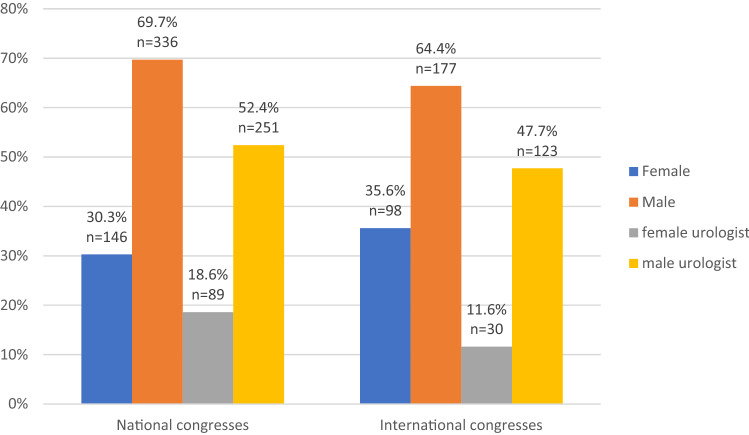
Gender distribution of female chairs by congress type

There was no statistically significant difference in the chair gender distribution compared between national and international congresses (*p = *0.130). Considering gender distribution by medical specialty, significant more women were invited outside of the urology community. Female urologist chairs were only represented by 24.1% in the congresses compared to balanced gender distribution (49% vs. 51%) if chairs were invited outside of the urology field (*p < *0.001, Fig. 1). When controlled for congress type, the gap for female urologists was more pronounced in national congresses compared to international congresses (Fig. 2). In contrast, distribution of female chairs outside of the urology field was varying between the congress type, whereas 61% were represented in international and 40% in national congresses. Mantel–Haenszel statistics could not be applied due to the described heterogeneities of the group distributions.

Countries with more than 80% male chair representation were Turkey [*n = *48 (84.2%)] and Belgium [*n = *5, (83.3%)]. Countries with less than 60% male chair representation were Sweden [*n = *36 (59.0%)], United Kingdom [*n = *9 (45%)] and the United States [*n = *10 (43.5%)].

The majority of affiliations were university 480 (63.3%) and community hospitals 232 (30.6%) with no significant difference between affiliation and gender distribution (*p = *0.059, 68% men vs. 32% women for university and community hospitals, respectively). No association between specific session topics and gender could be identified (*p = *0.460). However, significant differences in session type and gender could be identified (*p < *0.001, Table 1), whereas most men were invited to plenary sessions (73.2% vs. 26.8%) compared to abstracts sessions (59.0% vs. 41%), Workshops (67.1% vs. 32.9%) or Video sessions (64.7% vs. 35.3%).

**Table 1:** Percentual Gender distribution of speakers by session type

Session type	Gender	
	Male, *n* (%)	Female *n* (%)
Abstract	354 (26.7)	268 (33.9)
Plenary/Podium	556 (41.9)	274 (34.6)
Video	11 (0.8)	7 (0.9)
Workshop	405 (30.5)	242 (30.6)

### Speaker analysis

A total of 2135 speakers were identified in functional and female urology topics, which were distributed by 988 (46.3%), 397 (18.6%) and 750 (35.1%) for the years 2019, 2020 and 2021. Gender distribution was 1326 (62.1%) and 790 (37.0%) for male and women, respectively, and 19 (0.9%) missing gender information. No significant differences in gender distribution between the years could be identified (*p = *0.367). This represents a gender gap of 25.1% for speaker.

A total of 996 (46.6%) and 1139 (53.3%) speakers were identified in international and national congresses, respectively. There was no difference in gender distribution compared between national (61.6% male vs. 38.4% female) and international (63.6% male and 36.4% female) congresses (*p = *0.355). Regarding session type, significant more male speakers were invited to plenary sessions compared to other types and furthermore, abstract sessions were dominated by female speakers (*p < *0.001, Table 1).

Countries with more than 80% of male speaker representation were Turkey [*n = *84 (88.4%)] and Portugal [*n = *46 (85.2%)], whereas countries with less of 60% male speaker representation were Sweden [*n = *155 (59.4%)], the United Kingdom [*n = *33 (58.9%)] and Italy [*n = *121 (47.3%)].

## Discussion

The current investigation aimed to evaluate the gender representation at functional urology and female urology sessions in international and national urologic academic meetings. On assessment of chair and speaker representation, we identified an overall gender gap of 28%. This gap was more pronounced in gender representation of chairs of sessions with a gap of 35.5%. Interestingly, considering only chairs who were invited originated from outwith the urology field, a balanced gender representation was achieved. By contrast, female chairs invited from the urology field were underrepresented by 24%. Furthermore, in terms of the gender gap for speakers was 25%. Plenary/Podiums sessions were uniformly dominated by male in both, chair and speaker categories, compared to other session types. No differences of gender representation of chairs and speakers between national and international congresses could be identified in functional urology. Interestingly, less than 60% male representation for chairs and speakers were consistently identified in Sweden and United Kingdom.

This study confirmed the existing gender gap for functional urology sessions. A comparable analysis of the North American Neuromodulation Society meeting identified similar results, although a steadily increase of female speaker representation over the last 5 years was observed [10]. Nevertheless, female representation in the field of functional urology is notably higher when compared to urology congresses including all oncology and non-oncology topics in Europe and the United States [4, 8, 11]. The gender gap a large European urology congress was recently reported as 65% [4], which represents a gender gap difference compared to the current study focusing only on functional urology topics of 37%.

Women are often choose to go into functional urology based on stereoptypical pereception based on gender [12, 13]. This has been confirmed in a survey, where female responders felt that they were expected to choose functional urology over other subspecialities [13]. However, the majority of these women also reported, that they chose functional urology based on their own interest in the subspeciality [13]. A tendency of women to be interested in functional urology and external expectations could explain the higher female representation in functional urology compared to urology in general. Considering our results, there is higher female representation in functional urology. However, gender equity is also not achieved despite of having higher female numbers.

Furthermore, male speakers and chairs were preferably invited for plenary and podiums sessions. This finding is consistent with the literature and is a recognised problem in academia in general [4, 7]. A complete lack of female plenary speakers has been reported in up to 42.9% of surgical disciplines [7]. Interestingly, comparing the invitations for female chairs from within and outwith the urology discipline, the proportion of women invited outside the urology field was 49% whereas the proportion of women within the urology field was only 24%. This might be referred to as the “pipeline problem”. The pipeline problem is a common explanation that suggest that gender inequality will decrease once there is an adequate number of qualified women [14]. However, it has been demonstrated that gender inequity often persists despite the increase of females in academia. An American investigation on academia demonstrated that female associate professors constituted 10.9% but only 7.2% of full professor. In contrary, male associate professors constituted 16.4% but 28.1% were full professors. Furthermore, women earned less money in comparable positions. This disparity cannot be explained by a lack of qualified women or other reasons, rather than persistent systemic discrimination [14].

It has been demonstrated, that female scientists with identical qualifications and experience are judged to be less competent than their male counterparts, receive less mentoring offers and have lower starting salaries [15]. Male professors are almost twice as likely to have research discussions when conversing with male colleagues as compared to when conversing with a female colleague. Furthermore, women were described as sounding less competent during these discussions [2].

The increase of female representation depends on the individual societies efforts to aim diversity. Whereas some did not demonstrate any change of female representation over a 5 year period, others included, amongst others implementations, women in the program committees and achieved higher female representation [7]. In this study, the UK and Sweden have been identified as having the highest female representations at functional urology sessions. This might be the result of a conscious policy to include more women in scientific congresses. However, these results should be interpreted with caution. The number of chairs and speakers between the countries varied significantly and could not be used to answer the research question of gender representation at urology functional urology sessions between different countries.

The inclusion of women into the program committees has been identified as crucial factor for increasing female representation at conferences [2, 7, 16–18]. The average increase of female speakers for each additional female program committee member has been reported between 70 and 95% [2]. However, this represents only one aspect for achieving gender balance at scientific conferences. Ten simple rules have been established for achieving gender speaker balance [17]. Transparency in the form of establishing and publishing the conference speaker policies, as well as a family friendly realization of the conference are additional crucial steps [17].

On the other hand, women also have an important part to play. When women are invited for academic conferences, they should accept [17]. Women have been identified as more likely to decline invitations to conferences, to seek out shorter as opposed to more extensive talks and to ask less question than men [3]. Although this finding might be due to several factors, by being conscious of the importance of female representation at conferences, women can actively contribute to increase this number and change current practices.

We acknowledge some limitations of the current study. Chairs and speakers varied between the included countries limiting the comparative analyses. Furthermore, gender could not be or not clearly identified in some cases, although this number was low. Comparative analysis between countries was limited due to imbalanced number conferences included per country.

Furthermore, this study addressed only one of the factors potentially involved in discrimination, which is gender. Other possible sources of discrimination exist including identity, sexual orientation, cultural practices, ethnicity and race [9, 19]. Traditional under-represented groups are confronted with less career opportunities which includes particularly women, but also individuals living in low-middle income countries and racialized persons [20]. An analysis of gender and racial representation at US abdominal radiology conferences identified a trend for increasing female gender representation whereas racial representation remained persistently low. The majority of plenary speaker were white and no speaker was Black, African American or Native Americans at all [21]. Further studies will need to address these other factors of exclusion. Finally, female representation in functional urology field has not been evaluated for Asia. Evidence on female representation in academic conferences in Asia are sparse and no evidence for functional urology could be found at all. This result could be limited due to English language search. However, gender inequity in leadership positions in medicine is also evident in Asia [22]. These refers to several explicit and implicit biases, including traditional beliefs about the role of women in households. Furthermore, Asian societies tend to favor obedience over individual growth which increases female challenges in a professional career [22]. Further studies are needed to investigate female representation in Asian conferences which has not been sufficiently addressed, yet.

## Conclusions

Academic conferences are a crucial gateway to an academic career [1]. The numerical representation of women conferences is an important factor that influences the perception of individual women of the how inclusive the discipline is. Importantly, these perceptions differ substantially between men and women. In functional urology sessions, women are still underrepresented. However, compared to other subspecialities, functional urology has a substantial higher female representation. However, the number of female urologists was still limited. Guidance for achieving gender representation at academic conferences have been published. One of the most crucial factors are the inclusion of women into the program committee, transparency of the speaker policy and providing a family friendly environment at the congress.

Gender equality is our collective responsibility [23]. Female urologists must be actively supported by programs [24], provided by the highest departmental and institutional levels to herald a permanent change [25].

## Data Availability

Research data are not shared.
